# 

**Published:** 1873-06

**Authors:** 


					CONTENTS:
ORIGINAL COMMUNICATIONS.
Georgia State Dental Society.
Article I.?
The Action of Cohesive Foil under the Instrument.
By E. L. Hun
eri
Article II.
SELECTED ARTICLES.
-Furrowed Enamel in Connection with Syphilitic and oilier Kxh
Disease?.
By S. P. Cutler, M. D.
Article III.-
-Mixtures of Ether and Chloroforni^-The Vienna Mixture
Article IV.-
?Effect of Nitrous Oxide Gas..
Article V.?
Amputation of Part of the Tongue (or Ciirciuomatous Disease.
Article VI.?
Effects of Alcoholism Upon the Nervous System.
Article VIL?
-Surgery.
Article VIII.-
...w.
Testimony in Favor of Dr. Arthur's Method.
Bvllieo. F. Oiupein, DD
Article IX.-
?On the Treatment of Fraciure of the Inferioi Maxilla.
By D. R. Silver,
Article X.-
New Method^ot 1 reating Ulcers.
Article XI.-
-Electricity as a Mt:;ins of Resuscitation.
Article XII.-
A New Specific for Rheumatism
Article XIII.
EDITORIAL DEPARTMENT.
A Cause of Facial Neuralgia
Removal of Cystic Tumors from the Eyelids.
Colorless Tincture of Iodine
The Southern Dental Association
American Dental Convention
MONTHLY SUMMARY.
Brain Stimulants
Labor
A Sure Test as to Whether Lift is Extinct or Not.
False Teeth
Dental Accidents During Anaesthesia
Professor Haeckel on the Origin of Man
Dangers of Chromic Acid
Glycerine Lotion
The Wonders of the Deep
Neuralgic Pill.

				

